# Classification of vertical jump performance categories in futsal using machine learning algorithms

**DOI:** 10.3389/fspor.2026.1852848

**Published:** 2026-07-01

**Authors:** Diana Ximena Martínez-Arce, Laura Andrea Quintero-Palma, Jessica Quiceno-Henao, Dario Cuasapud-Arroyave, Jhonatan Betancourt-Peña, Wilfredo Agredo-Rodríguez, Jesus Alfonso Lopez-Sotelo

**Affiliations:** 1 Laboratorio Integrado de Análisis del Movimiento, Institución Universitaria Escuela Nacional del Deporte, Santiago de Cali, Colombia; 2 Faculty of Engineering and Basic Sciences, Universidad Autónoma de Occidente, Santiago de Cali, Colombia; 3 Centro de Estudios Olímpicos, Institución Universitaria Escuela Nacional del Deporte, Santiago de Cali, Colombia

**Keywords:** biomechanics, classification algorithms, force plates, futsal, machine learning, vertical jump

## Abstract

**Background:**

Futsal requires high-intensity explosive actions, including vertical jumps. Force-time analysis from dual force plates provides multidimensional biomechanical data that traditional statistical methods struggle to analyze effectively.

**Objective:**

To develop and evaluate a supervised classification framework capable of reproducing internally-derived vertical jump performance categories in futsal athletes using independent force-time metrics from dual force plates.

**Methods:**

Fifty-one male athletes performed countermovement jumps (CMJ), squat jumps (SJ), and drop jumps (DJ) on VALD ForceDecks dual force plates (1,000 Hz), yielding 148 valid observations after exclusion of records with missing PCA-input variables. Four internally derived performance categories were constructed using Principal Component Analysis of six biomechanical variables. The first two retained components explained 75.6% of the total variance. We compared four machine learning algorithms using an approximate 75/25 athlete-level stratified group split and stratified 5-fold cross-validation with group constraints to prevent leakage from repeated jump records from the same athlete. Robustness was assessed through reduced-predictor sensitivity analysis, jump-test ablation, learning curves, calibration, and nested cross-validation.

**Results:**

Logistic Regression achieved strong performance on the independent test set (F1-Score = 0.830, AUC-ROC = 0.977). Grouped 5-fold cross-validation yielded more conservative estimates (F1 = 0.770 ± 0.069, AUC = 0.941 ± 0.039), reflecting a more realistic estimate of internal generalization to unseen athletes. Coefficient-based and permutation importance analyses consistently identified CMJ peak power as the most influential predictor, while DJ-derived variables, particularly eccentric mean force, concentric impulse, and peak power, also showed substantial discriminative relevance. Ablation analysis confirmed the critical role of drop-jump variables: removing them reduced F1-score from 0.830 to 0.604, corresponding to an absolute decrease of 0.226 and a relative reduction of approximately 27%.

**Conclusions:**

Machine learning algorithms, particularly Logistic Regression, supported accurate classification of internally derived PCA-based vertical jump performance categories in futsal athletes. The combination of PCA-based category construction and supervised classification, with rigorous athlete-level validation and comprehensive robustness diagnostics, provides a reproducible methodological framework that may support talent identification, individualized training, and longitudinal monitoring after external validation.

## 1 Introduction

Futsal is a team sport characterized by high-intensity, intermittent physical demands, in which athletes execute multiple explosive actions, such as sprints, changes of direction, defensive and offensive jumps, and sudden decelerations, in confined spaces (1, 2). The ability to generate explosive power in the lower limbs constitutes a critical determinant of sports performance, correlating directly with the ability to execute decisive actions during competition (3). In this context, vertical jump assessment has become established as a recommended protocol for quantifying neuromuscular function, accumulated fatigue, and recovery capacity in elite athletes (4, 5).

Traditionally, vertical jump assessment has focused on discrete metrics, with jump height being the most frequently used variable in the scientific literature and in sports practice (6). While this metric provides useful information about explosive force production capacity, it simplifies a biomechanically complex movement involving multiple temporally coordinated phases (7). The underlying neuromuscular strategy shapes the multifactorial nature of jump performance and is reflected in the force-time curve during the eccentric and concentric phases (8, 9). Recent studies have demonstrated that variables derived from the force-time curve, such as the modified reactive strength index (RSI-mod) and peak power, may be more sensitive to changes in neuromuscular status than jump height alone (4, 10).

The technological evolution of dual force plates has transformed the capture of biomechanical data during jump assessments (8, 11). These systems provide a detailed view of ground reaction forces at 1,000 Hz, enabling the derivation of multiple kinetic and kinematic variables that characterize the different phases of the jump (12, 13). However, this wealth of information poses an analytical challenge: synthesizing multiple correlated variables into performance categories that are simultaneously coherent from a biomechanical perspective and applicable to coaches and sports scientists (14).

Traditional statistical methods, including analysis of variance and bivariate correlations, often face limitations when dealing with the high dimensionality and multicollinearity that characterize biomechanical data from force plates (15). These limitations result in fragmented interpretations that do not capture the integrated nature of neuromuscular performance, making it difficult to translate scientific findings into practical recommendations for training planning (16). The complexity of sports dynamics and the multidimensional aspects of athletic performance require more sophisticated analytical approaches that can handle multiple predictor variables simultaneously (15).

In this context, machine learning algorithms emerge as a promising analytical tool for addressing the inherent complexity of multidimensional biomechanical data (17). Specifically, supervised classification algorithms can classify biomechanical observations into discrete performance categories by simultaneously analyzing multiple predictor variables (18).

Recent research has demonstrated that models such as Random Forest can predict changes in countermovement jump performance with high accuracy, using pooled data from multiple meta-analyses (19). Within this growing field, interpretable machine learning frameworks applied to simulation-derived biomechanical features have demonstrated the capacity to identify task-specific muscular imbalances in running, highlighting the discriminative relevance of force-derived predictors and the value of model transparency for applied biomechanical assessment (20). However, the specific application of supervised learning to classify internally derived vertical jump performance categories based on force plate data remains relatively unexplored in professional and university futsal, particularly in Latin American contexts.

An additional consideration relevant to applied translation is technological portability. Several biomechanical variables used to construct performance categories can be obtained using accessible field technologies, including contact mats and validated jump-analysis applications, whereas the force-time predictor variables require dual force plates. This asymmetric design was intended to support the future transferability of the category framework to settings with limited laboratory equipment.

The present study develops and evaluates a two-stage analytical framework designed to construct internally derived PCA-based vertical jump performance categories in futsal athletes and to assess whether supervised machine learning algorithms can classify these categories using independent force-time predictors. This approach seeks to move beyond the simple quantification of vertical jump performance by integrating category construction, supervised classification, athlete-level validation, and robustness analyses. In applied settings, this framework may support training planning, fatigue monitoring, and sports rehabilitation, although external validation is required before broader implementation.

## 2 Methods

### 2.1 Study design

An observational, cross-sectional, and analytical study design was employed. The Research Ethics Committee of the Institución Universitaria Escuela Nacional del Deporte (Act No. 40.07.239) approved this study. All participants were informed about the procedures, potential risks, and benefits of the study, and signed informed consent prior to participation, in accordance with the principles of the Declaration of Helsinki.

The methodological process comprised five sequential stages: (1) biomechanical data acquisition from vertical jump tests (CMJ, SJ, and DJ) using dual force platforms; (2) data preprocessing including missing value verification, outlier handling, and *Z*-score standardization; (3) category construction through principal component analysis to define a multiclass target variable integrating performance level and mechanical strategy; (4) feature selection retaining predictor variables independent from those used in category construction to prevent data leakage; and (5) supervised classification model development with athlete-level train/test splitting and stratified cross-validation.

### 2.2 Participants

A convenience sample of 51 male futsal athletes, distributed across two competitive levels, was recruited: 27 professionals affiliated with teams from the Colombian Professional League and 24 university players on a representative team from a University in Santiago de Cali. The sample had a mean age of 24.0 ± 5.8 years and body mass of 73.18 ± 11.73 kg. All participants were over 18 years of age, attended at least three training sessions per week, and had no recent musculoskeletal injuries in the lower limbs that could compromise jump performance. We excluded athletes with acute pain at the time of assessment, and those with medical conditions contraindicated high-intensity exercise.

### 2.3 Experimental procedure

We conducted the assessments in two sessions within each team's usual training environment. The first session included anthropometric measurements and familiarization with the jump protocol. We conducted the vertical jump assessments in the second session. Before testing, athletes completed a standardized 10-min warm-up led by a coach. This protocol consisted of: low-intensity jogging, dynamic joint mobility exercises for the hip and knee, a moderate-intensity change-of-direction circuit, and submaximal jumps at 60% of perceived exertion.

The assessment protocol included three vertical jump modalities in the following order: countermovement jump (CMJ), squat jump (SJ), and drop jump (DJ). For each modality, athletes performed three valid maximal repetitions, with a 60-s rest period between attempts to ensure complete neuromuscular system recovery (5, 6). The CMJ was executed from an upright standing position, with hands on the iliac crests, performing a countermovement to approximately 90° of knee flexion, followed by a maximal jump (21). The SJ was performed from a semi-squat position, without countermovement, with hands on the waist before executing the maximal concentric jump (21). Athletes performed the drop jump (DJ) from a 30 cm platform, with instructions to minimize contact time and maximize rebound height (22).

We conducted the assessments using the VALD ForceDecks model FD4000 dual force plates (VALD Performance, Brisbane, Australia) at a sampling frequency of 1,000 Hz. This system allows simultaneous, independent capture of ground reaction forces for each lower limb, providing high-temporal-resolution data for jump-phase analysis. We acquired and initially processed the data using ForceDecks software, which uses validated algorithms to automatically detect jump phases (8).

From these assessments, biomechanical variables derived from the CMJ, SJ, and DJ tests were obtained, which were used for two complementary analytical purposes: a subset of variables was employed for the construction of internally derived PCA-based vertical jump performance categories, while another set comprised the predictor variables used in the training of classification models (Table 1).

**Table 1:** Description of the variables included in the study according to category, unit of measurement, and function.

Category	Variable	Unit of measurement	Function
Demographic	Age	years	Descriptive/excluded
Demographic	Body mass	kg	PCA Category Construction
CMJ	CMJ jump height	cm	PCA Category Construction
CMJ	CMJ modified RSI	m/s	PCA Category Construction
CMJ	CMJ contraction time	ms	Prediction
CMJ	CMJ eccentric mean power	W/kg	Prediction
CMJ	CMJ peak power	W/kg	Prediction
CMJ	CMJ eccentric peak force	N/kg	Prediction
CMJ	CMJ landing peak force	N/kg	Prediction
CMJ	CMJ take-off peak force	N/kg	Prediction
CMJ	CMJ concentric impulse	N·s/kg	Prediction
SJ	SJ jump height	cm	PCA Category Construction
SJ	SJ concentric mean power	W/kg	Prediction
SJ	SJ peak power	W/kg	Prediction
SJ	SJ concentric mean force	N	Prediction
DJ	DJ inverse contact time	1/s	PCA Category Construction
DJ	DJ RSI	dimensionless	PCA Category Construction
DJ	DJ jump height	cm	Prediction
DJ	DJ peak power	W/kg	Prediction
DJ	DJ concentric mean force	N	Prediction
DJ	DJ eccentric mean force	N	Prediction
DJ	DJ concentric impulse	N·s/kg	Prediction
DJ	DJ impact peak force	N	Prediction

CMJ, countermovement jump; SJ, squat jump; DJ, drop jump; RSI, reactive strength index. Variables classified as “PCA-based category construction” were used in the principal component analysis to construct the internally derived multiclass target categories. Variables classified as “Prediction” constituted the independent force-time predictors used to train the supervised machine learning classification models. Age was included only as a descriptive sample characteristic and was excluded from both PCA-based category construction and predictive modeling.

### 2.4 Data processing

We obtained 153 jump records across the three jump types and excluded 5 records due to missing SJ values, yielding 148 valid observations for analysis (3.3% missing data). We inspected all continuous variables using descriptive statistics, Q–Q plots, and outlier analysis. We winsorized values exceeding 3 standard deviations and standardized the selected variables for category construction using *Z*-scores.

### 2.5 Category construction

For the construction of internally derived PCA-based vertical jump performance categories, 148 valid jump observations were retained after excluding records with missing PCA-input variables. A subset of six biomechanical variables was selected based on theoretical relevance, acceptable multicollinearity diagnostics using variance inflation factor criteria (VIF < 5) (23), and potential transferability across field-based assessment technologies: body mass (kg), CMJ jump height (cm), CMJ modified RSI (m/s), DJ inverse contact time (1/s), DJ RSI, and SJ jump height (cm). The DJ inverse contact time was calculated as the reciprocal of the DJ contact time, so that higher values represented shorter ground contact time and greater reactive capacity. These variables were used exclusively for PCA-based category construction and were not included in the supervised predictor set, thereby reducing the risk of direct data leakage between category definition and model training.

We used principal component analysis to determine the relative contribution of each biomechanical variable to the construction of internally derived PCA-based vertical jump performance categories (24). Prior to PCA, all input variables were standardized using *Z*-scores to ensure comparability across different units of measurement. The obtained factor loadings reflected the weight of each variable in each principal component, with higher absolute values indicating greater contribution to the component structure. PCA suitability was assessed using the Kaiser-Meyer-Olkin measure and Bartlett's test of sphericity. The KMO value indicated acceptable sampling adequacy (KMO = 0.617), and Bartlett's test confirmed that the correlation matrix was suitable for dimensionality reduction (*p* < 0.001).

We retained two principal components to construct internally derived PCA-based categories. Together, these components explained 75.60% of the total variance (PC1: 48.34%; PC2: 27.26%). The first component (PC1) showed positive factor loadings for jump performance variables, including CMJ jump height, CMJ RSI-mod, SJ jump height, and DJ RSI, and a negative loading for body mass, indicating that this component represented the overall level of neuromuscular performance. The second component (PC2) showed prominent loadings for variables related to reactive capacity, particularly DJ inverse contact time and DJ RSI, indicating a predominant mechanical strategy dimension. In this framework, higher PC2 values indicated a more elastic/reactive strategy, whereas lower PC2 values indicated a more concentric strategy.

**Figure 1:**
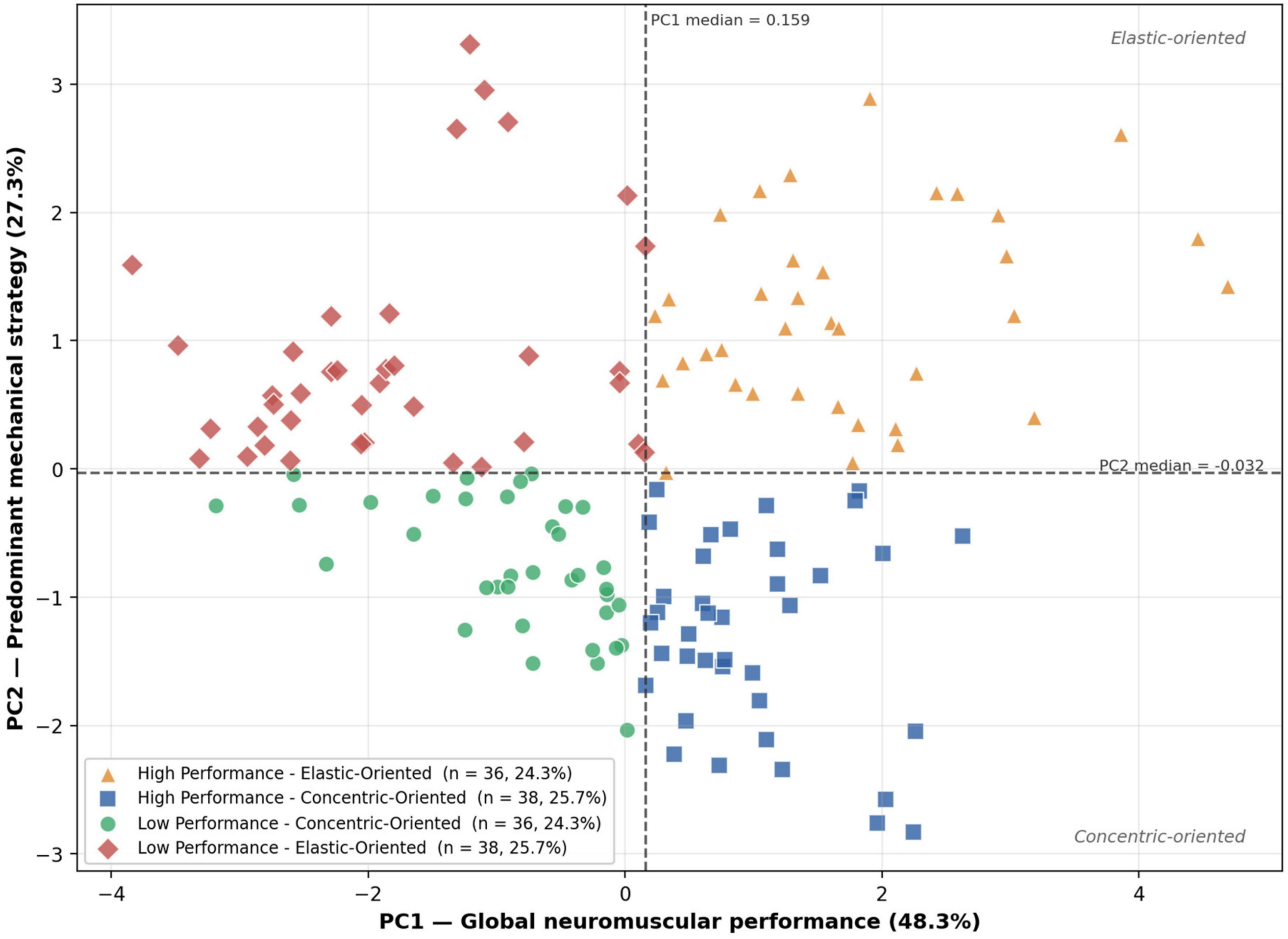
Distribution of the four internally-derived vertical jump performance categories in the PC1–PC2 space. PC1 represents global neuromuscular performance, and PC2 represents the mechanical strategy continuum. Dashed lines indicate the median-based thresholds (PC1 = 0.159, PC2 = −0.032).

The classification based on the median values of the retained principal components (PC1 = 0.159, PC2 = −0.032) produced a balanced distribution of records into four internally derived PCA-based vertical jump performance categories: High Performance—Elastic-Oriented Category (*n* = 36, 24.3%), High Performance—Concentric-Oriented Category (*n* = 38, 25.7%), Low Performance—Concentric-Oriented Category (*n* = 36, 24.3%), and Low Performance—Elastic-Oriented Category (*n* = 38, 25.7%). This categorization simultaneously integrates performance level, determined by PC1, and predominant mechanical strategy, determined by PC2. Within this analytical framework, elastic-oriented high-performance categories may represent a more favorable neuromuscular configuration for futsal-specific explosive demands, whereas low-performance concentric-oriented categories may indicate comparatively lower reactive capacity. These categories should be interpreted as internally derived, sample-dependent classifications rather than externally validated normative categories (Figure 1).

#### 2.5.1 Verification of operational independence

To address potential conceptual overlap between the PCA-derived target categories and the force-time predictors, we computed the full Pearson correlation matrix between the 16 predictor variables and the 6 PCA input variables, yielding 96 correlation pairs. The median absolute correlation was |*r*| = 0.278, the mean absolute correlation was |*r*| = 0.321, and 54.2% of the pairs showed low correlations (|*r*| < 0.30). However, 10 pairs (10.4%) showed high correlations (|*r*| ≥ 0.70), mainly between peak-power predictors and fundamental PCA-input variables, reflecting inherent biomechanical dependencies among variables derived from the same motor task. Accordingly, this study implements operational independence—because PCA-input variables were excluded from the supervised predictor set—but does not assume complete statistical independence among all vertical-jump biomechanical constructs.

#### 2.5.2 Sensitivity to alternative category construction

To evaluate the robustness of the median-based labeling strategy, we compared it with three unsupervised clustering approaches. The Adjusted Rand Index (ARI) values were 0.602 for K-Means, 0.404 for Gaussian Mixture Model, and 0.550 for Ward hierarchical clustering. These values indicate moderate agreement between the median-based labeling strategy and clustering alternatives, suggesting that the internally derived PCA-based categories reflected a partially recoverable latent structure but remained sensitive to the selected partitioning method. Bootstrap analysis of the median thresholds using 1,000 iterations yielded 95% confidence intervals of [−0.418, 0.605] for PC1 and [−0.493, 0.389] for PC2. These intervals indicate sample-dependent variability in the median cut-off points, which we acknowledge as a limitation due to the moderate sample size and the exploratory nature of the category-construction procedure.

### 2.6 Classification model development

To prevent data leakage, we excluded variables used in PCA-based category construction from the predictor set. The final classification model used 16 independent biomechanical variables as input features (X): CMJ contraction time (ms), CMJ eccentric mean power (W/kg), CMJ peak power (W/kg), CMJ eccentric peak force (N/kg), CMJ peak landing force (N/kg), CMJ peak takeoff force (N/kg), CMJ concentric impulse (N·s/kg), DJ jump height (cm), DJ peak power (W/kg), DJ concentric mean force (N), DJ eccentric mean force (N), DJ concentric impulse (N·s/kg), DJ peak impact force (N), SJ concentric mean power (W/kg), SJ peak power (W/kg), and SJ concentric mean force (N). The output variable (y) was a four-class categorical variable representing the internally derived vertical jump performance categories from PCA classification: High Performance—Elastic category, High Performance—Concentric category, Low Performance—Elastic category, and Low Performance—Concentric category.

We used Stratified Group K-Fold to split athletes into training and test sets, ensuring that no athlete appeared in both sets, maintaining class balance, and preventing data leakage from repeated measurements. This resulted in a training set of 38 athletes (*n* = 112 observations) and an independent test set of 12 athletes (*n* = 36 observations), with random_state = 42 to ensure reproducibility. To assess model stability and generalization capacity, stratified 5-fold cross-validation with group constraints was implemented over the full dataset, ensuring that athletes remained grouped across all validation folds. Model performance was quantified using accuracy, precision, recall, F1-Score (weighted average), and area under the ROC curve (AUC-ROC) with a one-vs.-rest multiclass strategy.

We evaluated four supervised classification algorithms in scikit-learn: Logistic Regression, Linear SVM, Random Forest, and RBF kernel SVM. For Logistic Regression, the solver was set to “lbfgs” due to its efficiency with multiclass problems, with max_iter increased to 5,000 to ensure convergence. A linear SVM was configured with *C* = 1.0 as the regularization parameter, with probability estimation enabled via Platt scaling. Random Forest employed 500 estimators with max_depth = None, allowing trees to expand until leaf purity, and parallel processing (n_jobs = −1) for computational efficiency. SVM with RBF kernel adopted gamma = “scale”, which automatically adjusts the kernel coefficient based on feature variance. All models incorporated class_weight = “balanced” to address the slight imbalance among performance categories. All preprocessing steps, including median imputation for missing values and StandardScaler, were encapsulated within scikit-learn Pipeline objects, with the scaler fitted only on training data within each cross-validation fold to prevent data leakage.

#### 2.6.1 Hyperparameter optimization and nested cross-validation

To verify that hyperparameter choices did not produce overly optimistic performance estimates, we implemented nested cross-validation using a 5-outer × 3-inner StratifiedGroupKFold design. Hyperparameter tuning was performed exclusively within the inner loop using a deliberately restricted grid, whereas the outer loop was used only for performance estimation. Athlete-level grouping was preserved in both loops to prevent leakage from repeated jump records belonging to the same athlete.

### 2.7 Model interpretability analysis

Model interpretability was planned for the algorithm showing the best balance between test-set performance, grouped cross-validation stability, calibration, and interpretability. For the selected model, feature importance was assessed using two complementary approaches. First, standardized model coefficients were extracted when available, and global importance was estimated as the mean absolute contribution across the four one-vs.-rest classifiers. Second, permutation importance was computed on the independent test set using 100 repeats and weighted F1-score as the scoring metric. This model-agnostic analysis complemented coefficient-based interpretation by estimating the decrease in predictive performance when each feature was permuted. Because correlated predictors can influence both coefficient-based and permutation-based importance estimates, feature importance was interpreted as relative predictive contribution rather than causal effect.

### 2.8 Reproducibility

A single global random seed (random_state = 42) was applied to stochastic procedures whenever supported by the corresponding algorithm. The analytical workflow was implemented using scikit-learn Pipeline objects to ensure that preprocessing steps, including imputation and standardization, were performed within the modeling workflow and did not introduce data leakage. Software versions and analytical outputs were documented to support reproducibility. The de-identified raw data are publicly available through a Figshare repository. The analytical code is not publicly available but may be provided by the corresponding author upon reasonable request, in accordance with institutional policies, journal requirements, and ethical data-use considerations.

## 3 Results

### 3.1 Classification model performance

Logistic Regression achieved strong performance on the independent test set, with an accuracy of 0.833, F1-score of 0.830, and AUC-ROC of 0.977 (Table 2). Linear SVM achieved the same test-set F1-score (0.830) but a lower AUC-ROC (0.957), while SVM with RBF kernel achieved an F1-score of 0.810 and AUC-ROC of 0.958. Random Forest showed the lowest test-set F1-score (0.615), despite maintaining an AUC-ROC of 0.930.

**Table 2:** Comparative performance of classification algorithms on the test set.

Algorithm	Accuracy	Precision	Recall	F1-Score	AUC-ROC
Logistic regression	**0.833**	**0.850**	**0.833**	**0.830**	**0.977**
Linear SVM	0.833	0.865	0.833	0.830	0.957
SVM RBF	0.806	0.851	0.806	0.810	0.958
Random forest	0.611	0.698	0.611	0.615	0.930

Bold values indicate the results for Logistic Regression, the model selected as the preferred algorithm based on its overall performance, stability, calibration, and interpretability.

Grouped 5-fold cross-validation provided more conservative and representative estimates of internal generalization to unseen athletes (Table 3). Under this evaluation, Logistic Regression achieved the highest mean F1-score and AUC-ROC (F1 = 0.770 ± 0.069; AUC = 0.941 ± 0.039), followed closely by Linear SVM (F1 = 0.768 ± 0.092; AUC = 0.934 ± 0.037) and SVM RBF (F1 = 0.750 ± 0.070; AUC = 0.934 ± 0.036). Random Forest showed lower and more variable performance (F1 = 0.715 ± 0.090; AUC = 0.915 ± 0.039). The difference between test-set and cross-validation performance reflects the variability introduced by differences in athlete composition across folds, and the grouped cross-validation estimates likely provide a more realistic assessment of internal generalization than a single test split. Nested cross-validation confirmed this conservative pattern: SVM RBF achieved the highest mean F1-score (0.752 ± 0.070), whereas Logistic Regression achieved a nearly equivalent F1-score (0.748 ± 0.090) and the highest AUC-ROC (0.932 ± 0.043). Given its comparable discrimination, better calibration, and greater interpretability, Logistic Regression was retained as the preferred model.

**Table 3:** Stratified cross-validation results (5 folds, StratifiedGroupKFold).

Algorithm	Accuracy (mean ± SD)	F1-Score (mean ± SD)	AUC-ROC (mean ± SD)
Logistic Regression	**0.779** **± 0.067**	**0.770** **± 0.069**	**0.941** **± 0.039**
Linear SVM	0.773 ± 0.093	0.768 ± 0.092	0.934 ± 0.037
SVM RBF	0.766 ± 0.057	0.750 ± 0.070	0.934 ± 0.036
Random Forest	0.724 ± 0.091	0.715 ± 0.090	0.915 ± 0.039

Bold values indicate the results for Logistic Regression, the model selected as the preferred algorithm based on its overall performance, stability, calibration, and interpretability.

**Figure 2:**
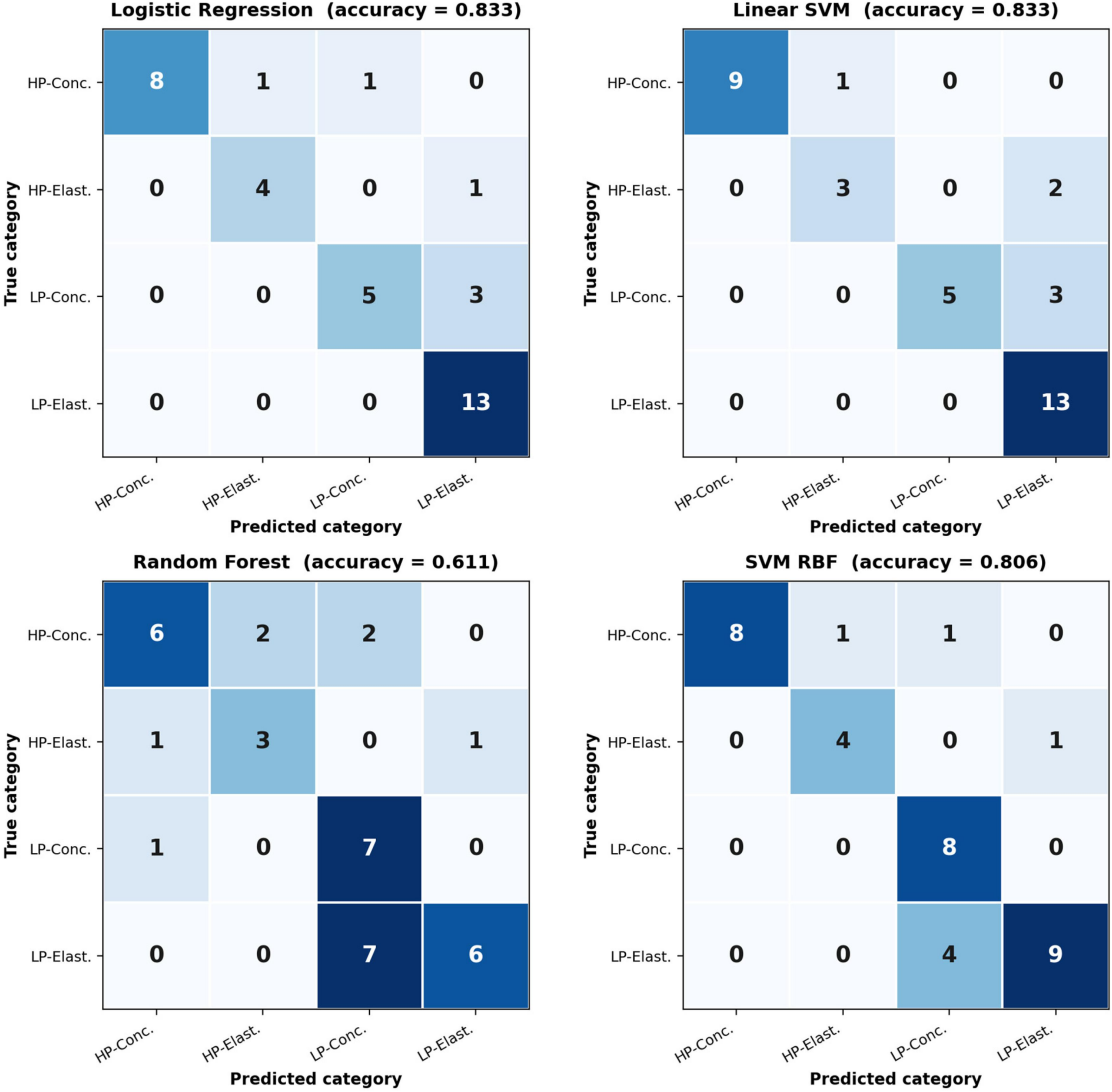
Confusion matrices for the four evaluated classification models on the independent test set (*n* = 36 observations). Across all models, misclassifications occurred primarily between categories sharing either a performance level or a mechanical strategy, suggesting error patterns aligned with the underlying biomechanical continuum. HP-Conc., high performance-concentric; HP-Elast., high performance-elastic; LP-Conc., low performance-concentric; LP-Elast., low performance-elastic.

The confusion matrices for the four evaluated models revealed coherent discrimination across the four internally derived categories (Figure 2). For Logistic Regression on the independent test set, the model correctly classified 8 of 10 High Performance—Concentric observations, 4 of 5 High Performance—Elastic observations, 5 of 8 Low Performance—Concentric observations, and 13 of 13 Low Performance—Elastic observations. Across all four algorithms, misclassifications occurred primarily between categories sharing either performance level or mechanical strategy, suggesting error patterns aligned with the underlying biomechanical continuum.

### 3.2 Robustness diagnostics

**Figure 3:**
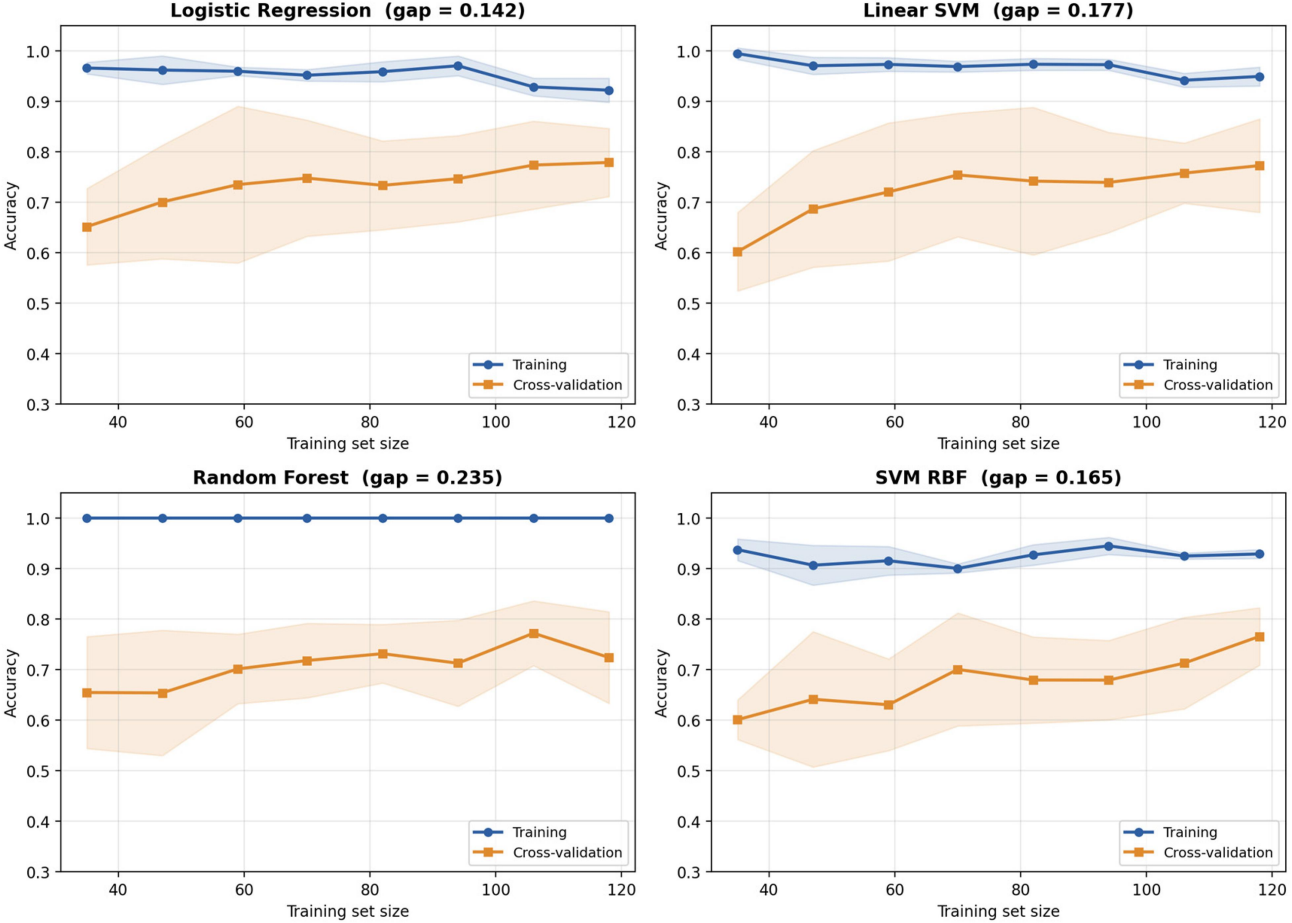
Learning curves for the four classification algorithms. Training and cross-validation accuracy are shown as a function of training set size. The final train–validation gap was smallest for Logistic Regression (0.142) and largest for Random Forest (0.235), suggesting a greater overfitting tendency for the tree-based model.

**Figure 4:**
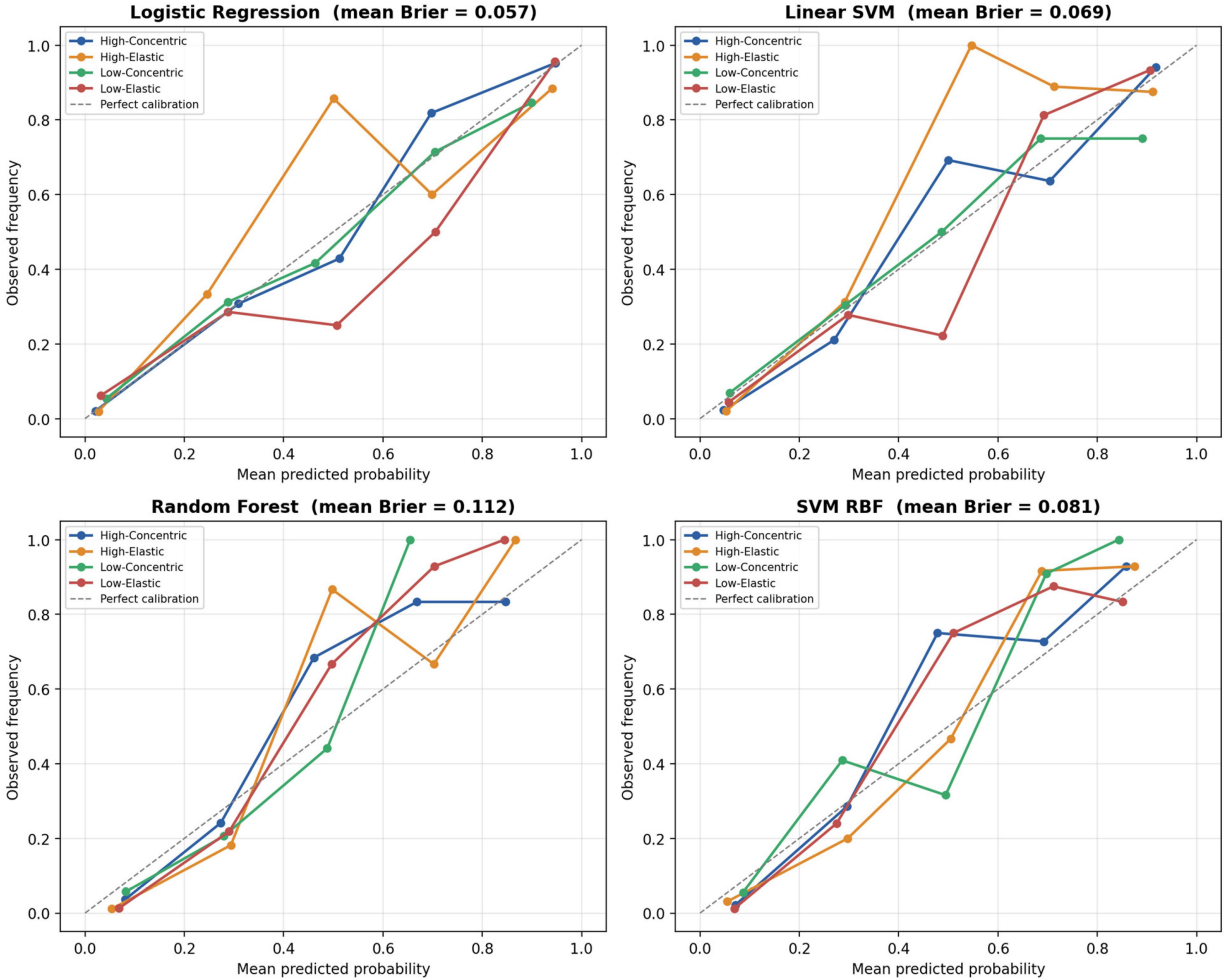
Multiclass calibration plots for the four algorithms. Logistic Regression achieved the best probability calibration (mean Brier Score = 0.057 across the four categories), indicating that its predicted probabilities were most consistent with observed frequencies.

The events-per-variable diagnostic yielded a classical EPV of 1.56 (112 training observations, 16 predictors, smallest class = 25). Although this value is below the conservative threshold of 10, recent methodological work has shown that EPV requirements are context-dependent and can be considerably lower for regularized, cross-validated prediction models (25, 26). Therefore, this diagnostic was interpreted as an indicator of the need for cautious interpretation rather than as a reason to discard the modeling approach. Learning curves indicated that Logistic Regression had the smallest train–validation gap (0.142), whereas Random Forest showed the largest gap (0.235), consistent with a greater tendency to overfit (Figure 3). Calibration analysis showed the best probability calibration for Logistic Regression (mean Brier Score = 0.057 across the four classes; Figure 4), suggesting that its predicted probabilities were more consistent with observed frequencies than those of the alternative models. Together, these diagnostics support selecting Logistic Regression as the preferred model and reinforce the need for external validation in larger, independent cohorts.

### 3.3 Sensitivity to category construction

**Figure 5:**
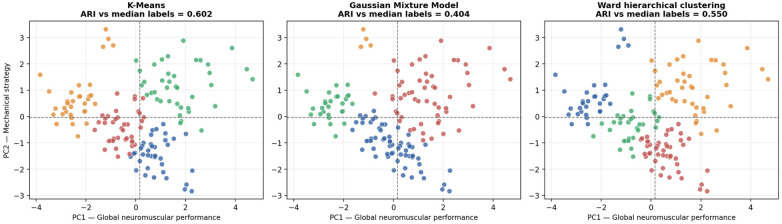
Comparison of the median-based labeling against three alternative unsupervised clustering strategies (K-means, Gaussian mixture model, ward hierarchical clustering) in the PC1–PC2 space. Adjusted Rand Index (ARI) values of 0.40–0.60 indicate moderate-to-substantial agreement, supporting the robustness of the median-based categorization.

The reduced-predictor sensitivity analysis excluded the seven predictors most strongly correlated with the PCA input variables (|*r*| ≥ 0.70). Under this conservative predictor set, Logistic Regression retained moderate discriminative capacity (F1 = 0.720; AUC = 0.910), although performance decreased relative to the full model. This finding indicates that highly correlated predictors contributed meaningful discriminative information, but that classification did not depend exclusively on them. Rather, the model preserved relevant classification capacity even when the most strongly overlapping predictors were removed. The comparison with alternative clustering strategies (Figure 5; ARI = 0.404–0.602) further supported the robustness of the median-based categorization and indicated sensitivity to the selected partitioning method.

### 3.4 Variable subset ablation

**Figure 6:**
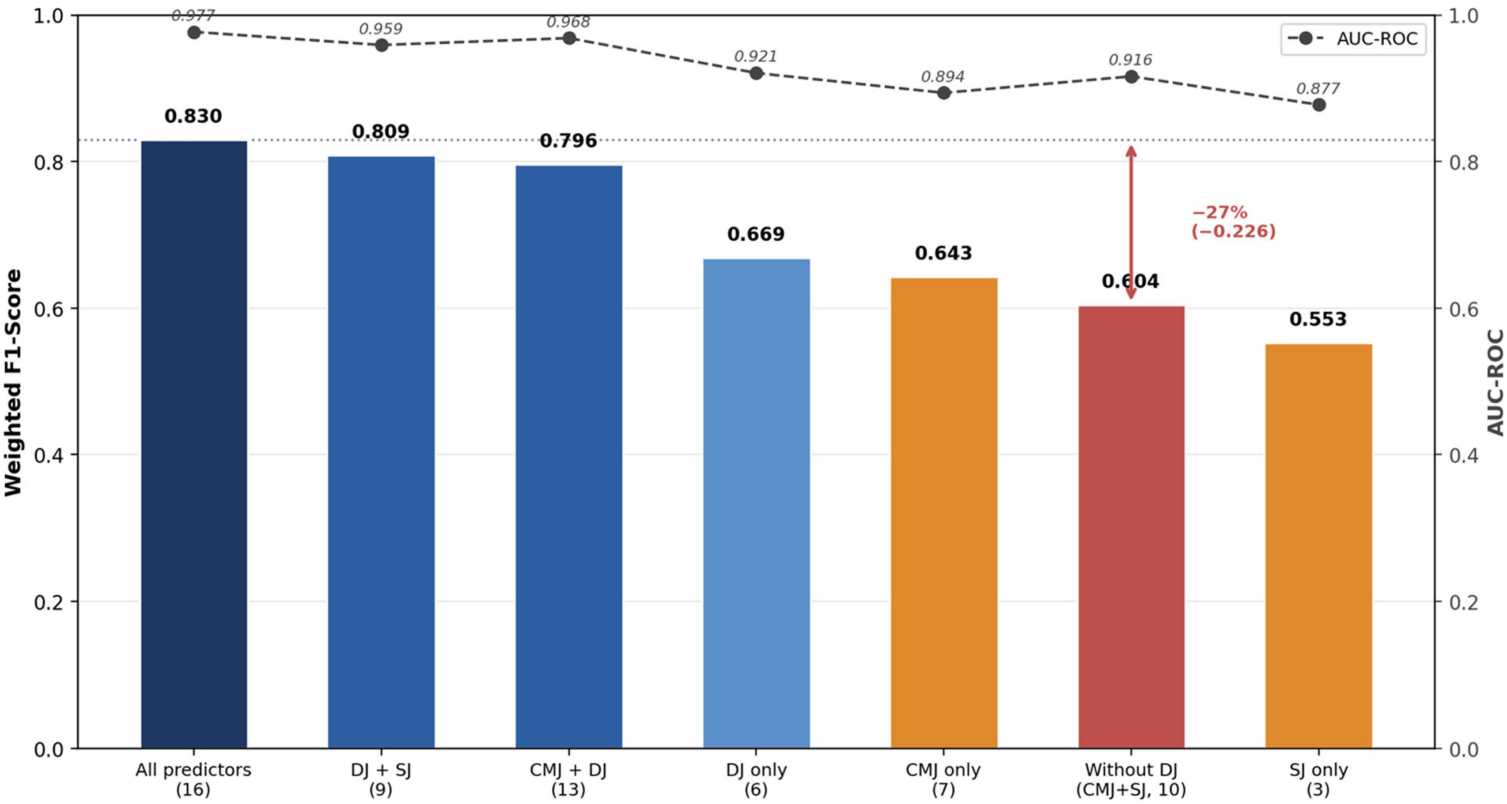
Variable subset ablation by jump test using logistic regression. The full model (16 predictors) achieved an F1 score of 0.830. Removing all drop-jump variables (“Without DJ”) reduced F1 to 0.604, a 27% relative reduction, confirming the central discriminative role of the drop jump.

Systematic ablation by jump test isolated the contribution of each jump modality (Figure 6). The full model using all 16 predictors achieved the best performance (F1 = 0.830; AUC = 0.977). Models including DJ-derived variables retained relatively high discriminative capacity, particularly DJ + SJ (F1 = 0.809; AUC = 0.959) and CMJ + DJ (F1 = 0.796; AUC = 0.968). In contrast, removing all DJ variables reduced the F1-score from 0.830 to 0.604, corresponding to an absolute decrease of 0.226 and a relative reduction of approximately 27%. Single-modality models showed lower performance, with DJ only achieving F1 = 0.669, CMJ only F1 = 0.643, and SJ only F1 = 0.553. These findings indicate that no single jump type provided sufficient discriminative capacity in isolation and that DJ-derived variables contributed substantially to the classification task.

### 3.5 Feature importance

Coefficient-based importance from the selected Logistic Regression model identified CMJ peak power (mean |*β*| = 0.881), DJ eccentric mean force (mean |*β*| = 0.847), DJ concentric impulse (mean |*β*| = 0.729), and DJ peak power (mean |*β*| = 0.643) as the most influential predictors. Permutation importance, a model-agnostic method, identified CMJ peak power (0.186 ± 0.057), SJ peak power (0.074 ± 0.043), and DJ eccentric mean force (0.067 ± 0.044) as the top predictors. Although the rankings were not identical, both methods consistently identified CMJ peak power and DJ eccentric mean force among the most relevant variables. DJ-derived metrics also occupied prominent positions across both approaches, supporting the discriminative value of reactive strength-related assessments. Because feature importance estimates may be influenced by correlated predictors, these findings were interpreted as relative predictive contributions rather than causal effects (Table 4).

**Table 4:** Feature importance ranking based on logistic regression OvR coefficients.

Variable	Mean |*β*|	Relative importance (%)	Permutation importance (%)
CMJ peak power	0.881	100.0	0.186 ± 0.057
DJ eccentric mean force	0.847	96.1	0.067 ± 0.044
DJ concentric impulse	0.729	82.7	0.011 ± 0.030
DJ peak power	0.643	73.0	0.062 ± 0.034
SJ peak power	0.576	65.4	0.074 ± 0.043
CMJ concentric impulse	0.571	64.8	0.014 ± 0.023
DJ peak impact force	0.502	57.0	0.026 ± 0.040
CMJ eccentric peak force	0.469	53.2	0.000 ± 0.027
SJ concentric mean force	0.433	49.2	0.023 ± 0.038
SJ concentric mean power	0.422	47.9	0.047 ± 0.030
DJ jump height	0.409	46.4	−0.001 ± 0.027
CMJ contraction time	0.370	42.0	0.028 ± 0.023
CMJ eccentric mean power	0.358	40.7	0.038 ± 0.039
DJ concentric mean force	0.226	25.7	−0.010 ± 0.025
CMJ peak landing force	0.182	20.7	−0.005 ± 0.026
CMJ peak takeoff force	0.081	9.2	0.009 ± 0.016

## 4 Discussion

The present study demonstrated that supervised classification algorithms can accurately classify internally derived PCA-based vertical jump performance categories in futsal athletes using biomechanical data from dual force plates. Before discussing the findings, we emphasize an important interpretive clarification: the four categories analyzed here are operational categories derived internally from the present dataset through median-based splitting of the first two principal components, not externally validated performance constructs. Therefore, the reported metrics reflect the classification framework's capacity to reproduce these internally derived assignments using independent force-time predictors, rather than to predict independent sport-performance outcomes.

Logistic Regression showed the best overall balance between test-set performance, grouped cross-validation stability, calibration, and interpretability. On the independent test set, Logistic Regression achieved F1 = 0.830 and AUC = 0.977, while grouped cross-validation yielded more conservative estimates (F1 = 0.770 ± 0.069; AUC = 0.941 ± 0.039). This difference between test-set and cross-validation performance reflects the variability introduced by athlete composition across folds and supports the use of grouped cross-validation as a more realistic estimate of internal generalization to unseen athletes. Nested cross-validation provided a further conservative evaluation: SVM RBF achieved the highest mean F1-score (0.752 ± 0.070), whereas Logistic Regression achieved a nearly equivalent F1-score (0.748 ± 0.090) and the highest AUC-ROC (0.932 ± 0.043). Given its comparable discrimination, superior calibration, and greater interpretability, Logistic Regression was retained as the preferred model.

From a practical standpoint, Logistic Regression offers several advantages, including enhanced interpretability through the direct examination of standardized coefficients, computational efficiency suitable for applied or field-based settings, and probabilistic outputs that may support confidence-weighted decision-making (16, 17). In contrast, Random Forest showed the largest train–validation gap in the learning-curve analysis, including perfect training accuracy (1.000) and lower validation performance. This pattern suggests greater susceptibility to overfitting in the present moderate-sized dataset, likely due to the greater flexibility of tree-based models when applied to limited grouped observations.

The interpretability analysis showed that CMJ peak power and drop-jump variables—particularly DJ eccentric mean force and DJ concentric impulse—were among the most influential predictors. CMJ peak power and DJ eccentric mean force ranked among the top predictors under both coefficient-based and permutation-based methods. This finding highlights the discriminative value of explosive power and reactive strength-related variables for differentiating internally derived PCA-based vertical jump performance categories, consistent with current evidence emphasizing the relevance of jump-derived force-time variables in performance monitoring and applied athlete categorization (4, 10). This finding also aligns with recent interpretable machine learning studies in sports biomechanics, where task-specific biomechanical predictors derived from motor tasks retained strong discriminative capacity under explainable modeling frameworks, further supporting the translational value of feature importance analyses in applied sports science contexts (20). The ablation analysis reinforced this interpretation: removing all DJ-derived variables reduced the F1-score from 0.830 to 0.604, corresponding to a relative reduction of approximately 27%. This indicates that the stretch-shortening cycle characteristics captured during DJ execution provide information that is not fully redundant with CMJ or SJ assessments (8, 9). Although practitioners often prioritize the CMJ because of its simplicity, widespread use, and reliability (5, 6), DJ assessment appears to provide substantial additional value for comprehensive neuromuscular classification in this framework (11, 22).

A relevant methodological contribution of this study was the implementation of athlete-level grouped data splitting, which ensured complete separation between training and test athletes. This approach addresses a common limitation in sports biomechanics research: multiple observations from the same athlete may appear in both training and validation datasets, leading to optimistically biased performance estimates due to within-athlete dependence (15, 17). The proposed classification framework may offer several practical applications after external validation: objective athlete categorization through reproducible classifications (14, 16); targeted training prescription, where athletes in lower-performance categories may benefit from progressive strength and plyometric training development (1–3); sensitive longitudinal monitoring through probabilistic outputs (4, 5); and optimization of assessment protocols by prioritizing DJ-derived variables and CMJ peak power when time or resources are limited (6, 8).

### 4.1 Limitations

Several limitations should be considered when interpreting these findings. First, the four vertical jump performance categories were internally derived operational categories based on PCA and median-based thresholds, not externally validated performance constructs. Future studies should validate these categories against independent criteria such as competitive performance, coach ratings, injury history, or longitudinal training response. Second, the sample was limited to 51 male futsal athletes from a single geographic region, restricting generalizability to female athletes, other age groups, different competitive levels, and other sports. Third, the cross-sectional design precludes assessment of category stability over time or sensitivity to training-induced adaptations. Fourth, the sample size yielded a low events-per-variable ratio (EPV = 1.56), and although regularization, grouped validation, calibration, learning curves, and nested cross-validation were used to characterize model stability, larger samples are required. Fifth, PCA adequacy was acceptable but moderate (KMO = 0.617), and bootstrap analysis indicated sample-dependent variability in the thresholds for PC1 and PC2. Finally, although direct data leakage was reduced by excluding PCA-input variables from the predictor set, residual biomechanical overlap remained because several variables were derived from the same jump tasks. External validation in an independent cohort is therefore the most important next step before broader implementation.

## 5 Conclusions

This study developed and internally validated a two-stage analytical framework for classifying internally derived PCA-based vertical jump performance categories in futsal athletes using force-time variables from dual force plates. The first stage used PCA to construct four operational categories that integrated global neuromuscular performance and predominant mechanical strategy, while the second stage evaluated whether supervised machine learning algorithms could classify these categories using independent biomechanical predictors.

Logistic Regression showed the best overall balance between test-set performance, grouped cross-validation stability, calibration, and interpretability. Although SVM RBF achieved a slightly higher F1-score in nested cross-validation, Logistic Regression maintained comparable discrimination, the highest AUC-ROC, better probability calibration, and greater interpretability, supporting its selection as the preferred model.

The interpretability and ablation analyses indicated that CMJ peak power and DJ-derived variables, particularly DJ eccentric mean force, DJ concentric impulse, and DJ peak power, contributed substantially to classification performance. These findings suggest that explosive power and reactive strength-related metrics provide complementary information for differentiating internally derived vertical jump performance categories.

From an applied perspective, this framework may support athlete categorization, training individualization, fatigue monitoring, and assessment protocol optimization in futsal. However, the categories should be interpreted as internal, sample-dependent operational classifications rather than externally validated performance constructs. External validation in independent cohorts remains the most important next step before this framework can be recommended for broader practical implementation. Future prospective studies should target sample sizes sufficient to meet established EPV requirements, follow contemporary reporting guidelines for artificial intelligence prediction models, and include populations beyond the present sample, particularly female athletes, other age groups, and distinct competitive contexts. Longitudinal and criterion-based validation designs would further strengthen the evidence base by assessing category stability over time and correspondence with independent performance outcomes. Until external validation is achieved, the present framework should be interpreted as internally validated and hypothesis-generating, providing a methodological foundation for future confirmatory research in sports biomechanics.

## Data Availability

The datasets presented in this study can be found in online repositories. The names of the repository/repositories and accession number(s) can be found below: https://figshare.com/s/ef8523361890da8ed2dd.
