# Supplementary material for: Classification of vertical jump performance categories in futsal using machine learning algorithms
Source: Front Sports Act Living. 2026 Jul 1;8:1852848. doi: 10.3389/fspor.2026.1852848 (PMC13371434; doi:10.3389/fspor.2026.1852848)
Supplement: Supplementary file 1 [file Datasheet1.docx]

Supplementary Material

# Supplementary Figures and Tables

## Supplementary Figures


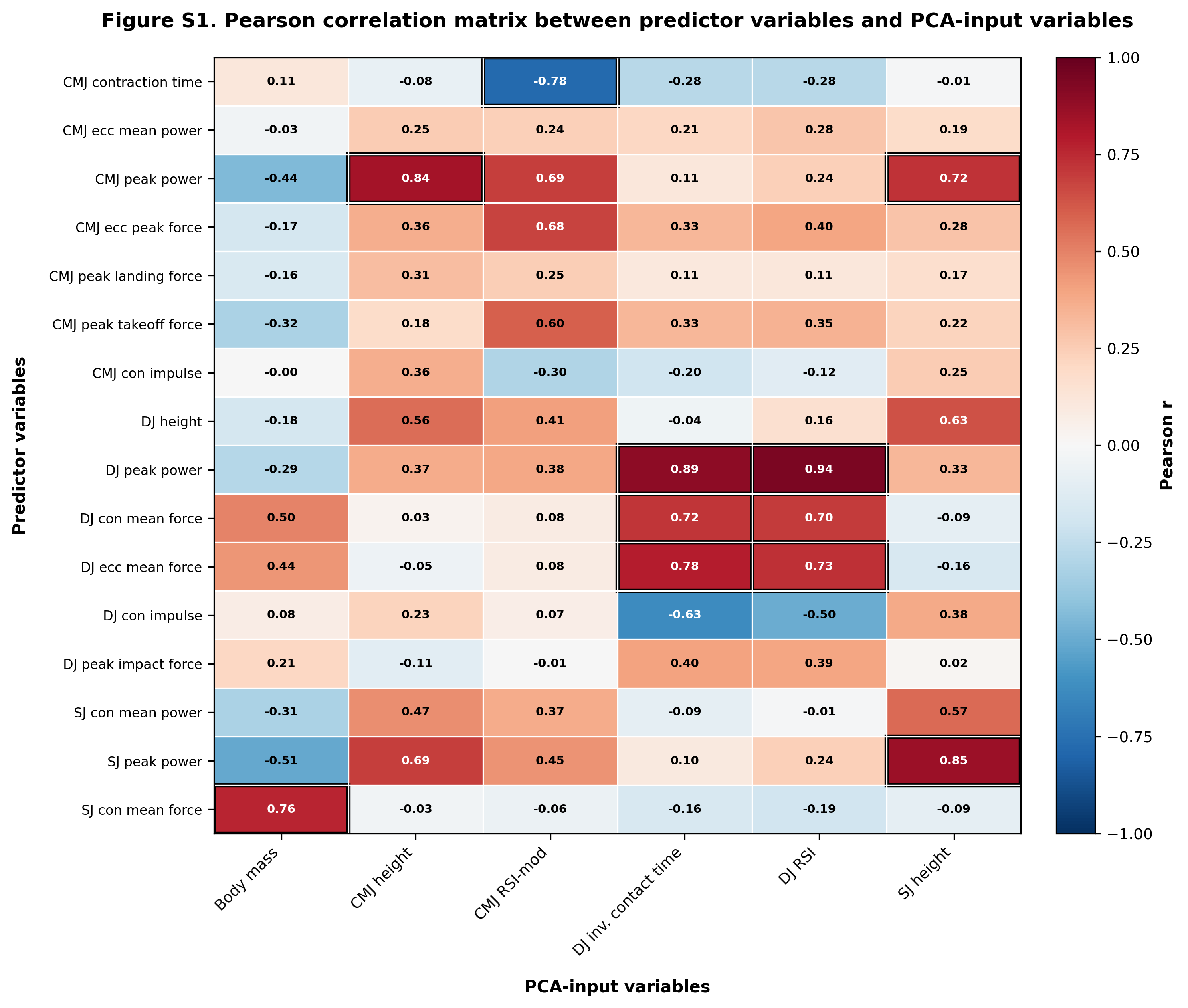
 **Supplementary Figure S1.** Pearson correlation matrix between the 16 force-time predictor variables and the 6 PCA-input variables (96 pairs). The median absolute correlation was |r| = 0.278, and only 10 pairs (10.4%) exceeded |r| ≥ 0.70 (outlined cells), confirming the operational independence between the predictor set and the variables used for category construction.


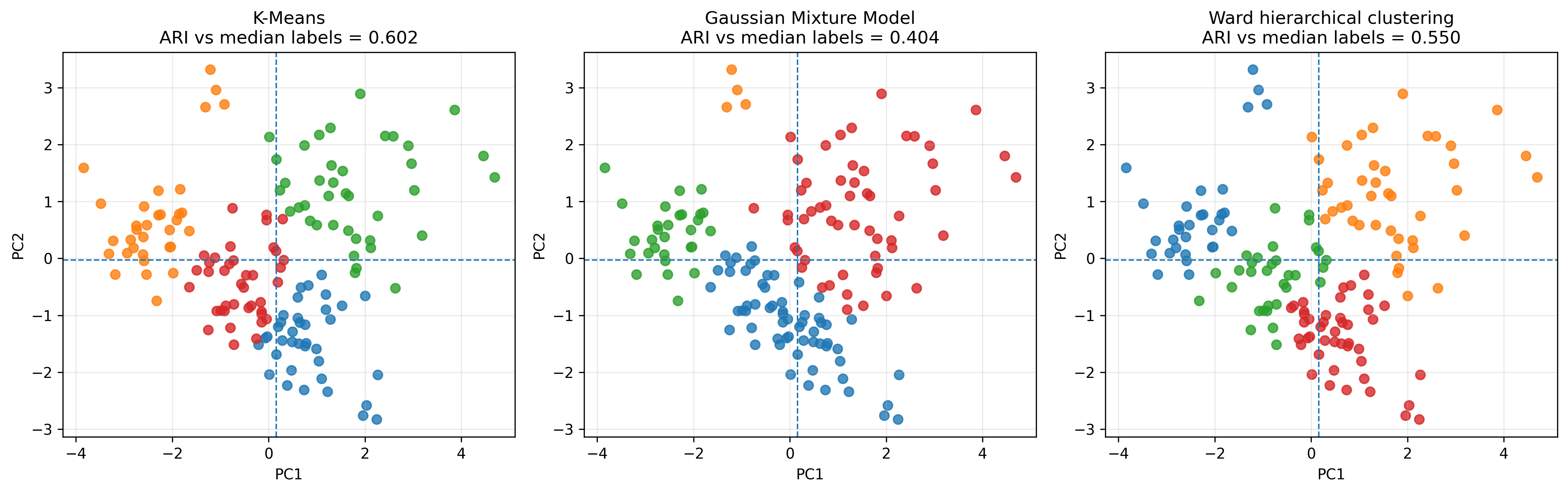


**Supplementary Figure S2**. Comparison of the median-based labeling against three alternative unsupervised clustering strategies (K-Means, Gaussian Mixture Model, Ward hierarchical clustering) in the PC1–PC2 space. Adjusted Rand Index (ARI) values of 0.40–0.60 indicate moderate-to-substantial agreement, supporting the robustness of the median-based categorization.


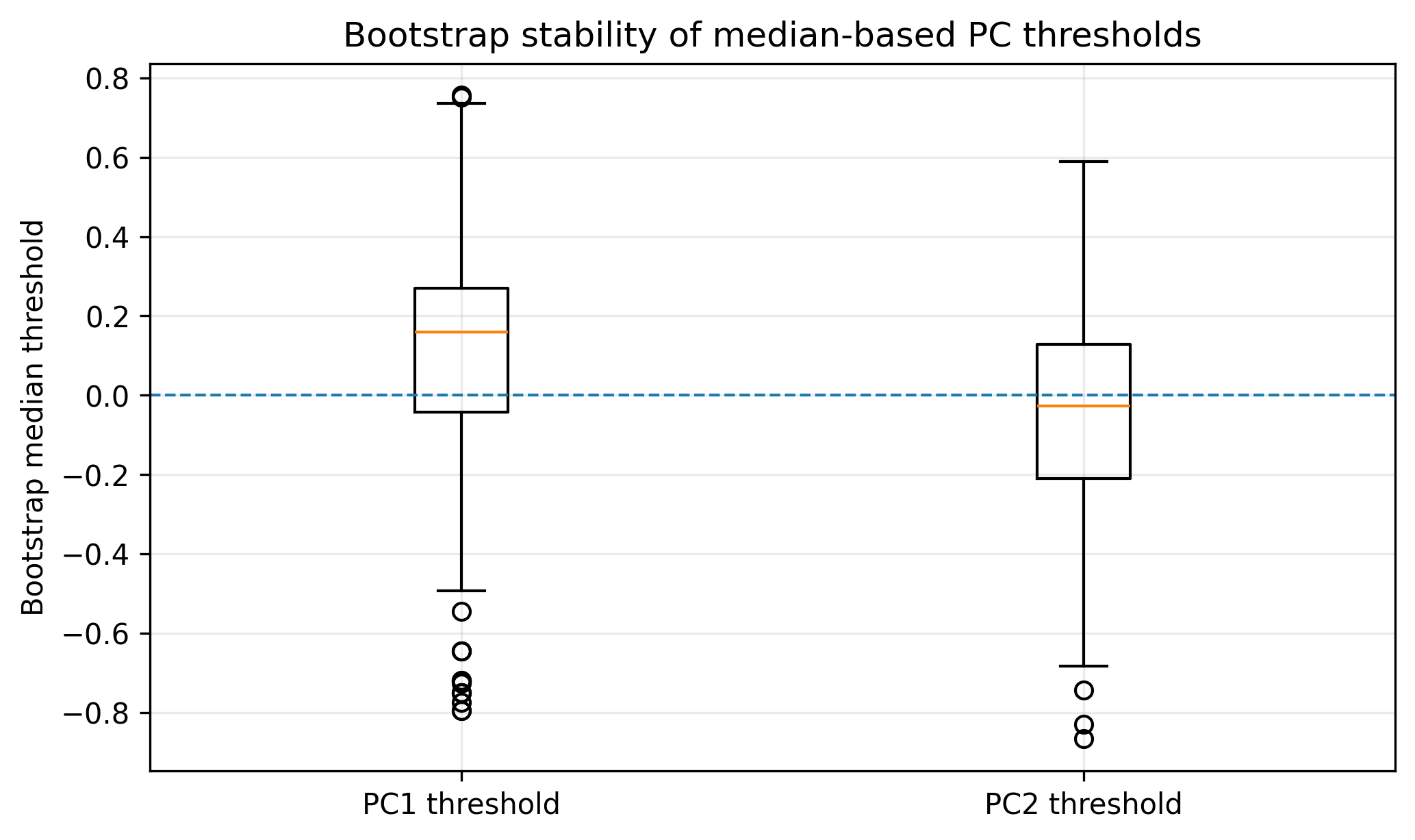


**Supplementary Figure S3.** Bootstrap distribution of the median thresholds for PC1 and PC2 across 1000 iterations. The 95% confidence intervals ([-0.418, 0.605] for PC1; [-0.493, 0.389] for PC2) indicate sample-dependent variability in the cut-off points, a limitation acknowledged due to the moderate sample size.


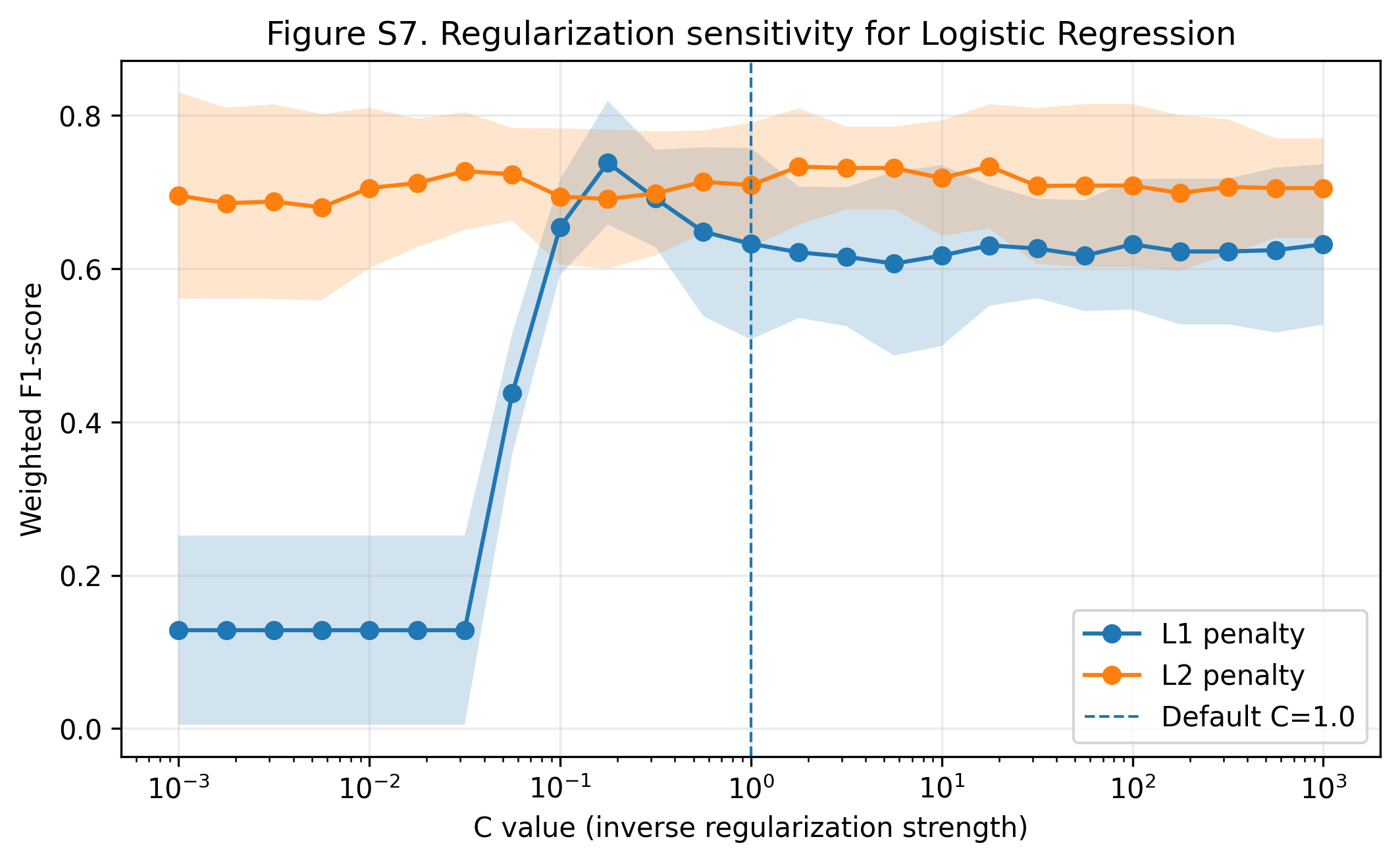


**Supplementary Figure S4.** Regularization sensitivity for Logistic Regression across a range of C values for L1 and L2 penalties. Performance was stable around the default C = 1.0 (dashed line), confirming that the default regularization was near-optimal and did not require aggressive tuning.


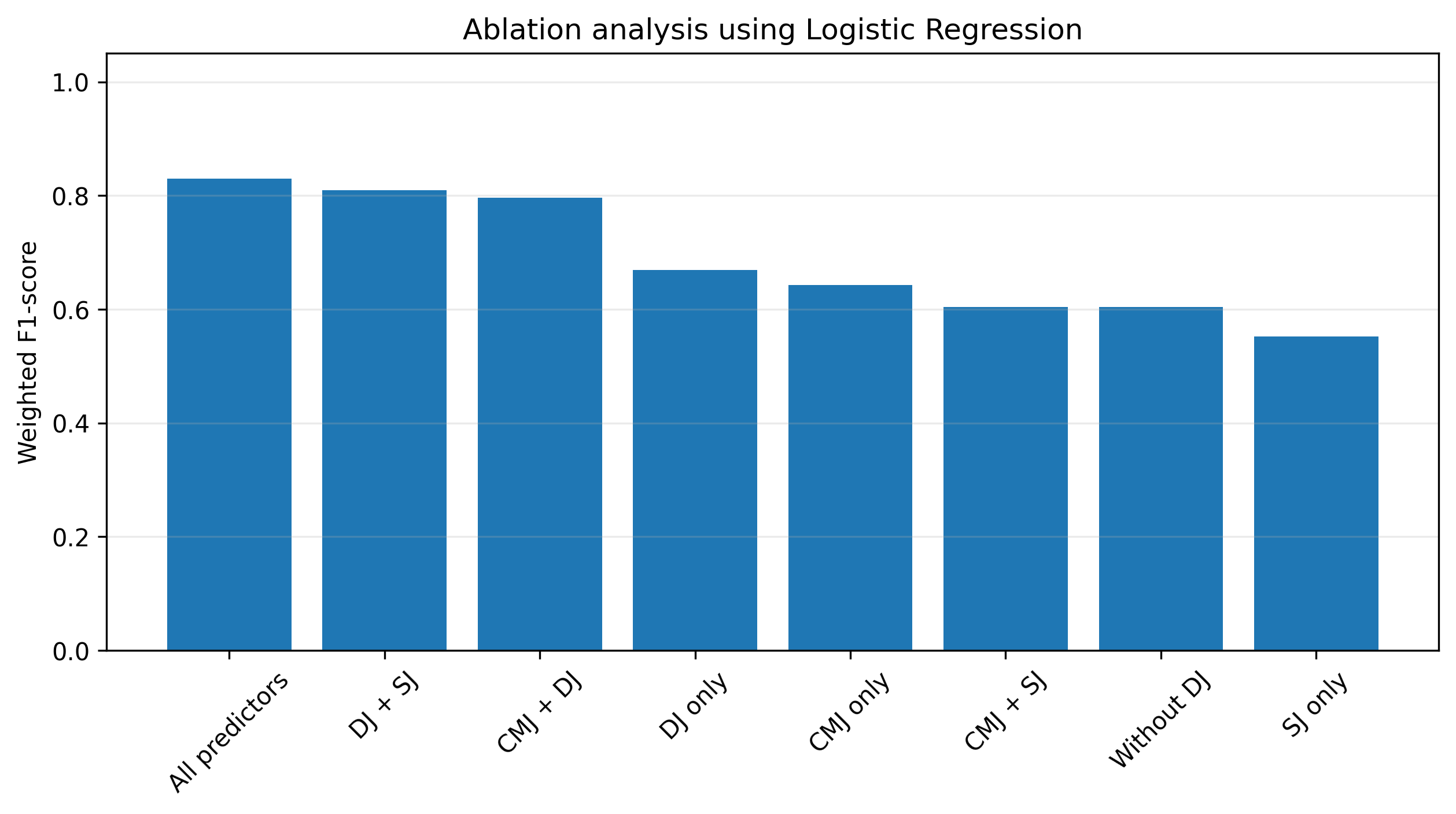


**Supplementary Figure S5.** Variable subset ablation by jump test using Logistic Regression. The full model (16 predictors) achieved an F1 score of 0.830. Removing all drop-jump variables (“Without DJ”) reduced F1 to 0.604, a 27% relative reduction, confirming the central discriminative role of the drop jump.


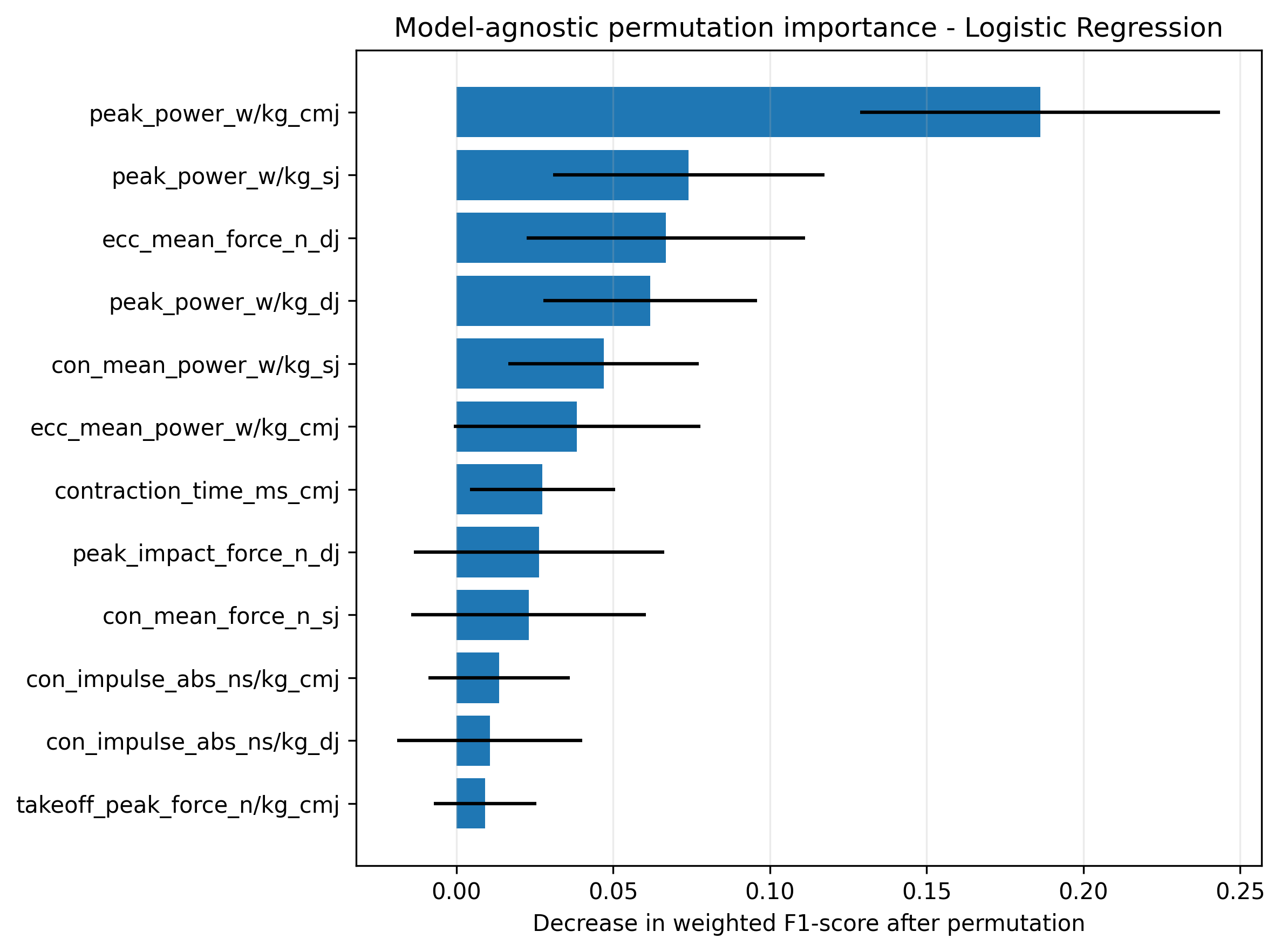


**Supplementary Figure S6.** Permutation importance of the predictor variables (n_repeats = 100 on the test set, weighted F1 scoring). CMJ peak power was the most influential predictor, followed by SJ peak power and DJ eccentric mean force, converging with the coefficient-based importance reported in the main manuscript. Error bars represent the standard deviation across permutations.

## Supplementary Tables

**Supplementary Table S1.** Variance explained by the principal components retained for category construction. The first two components were retained based on the Kaiser criterion (eigenvalue > 1) and a cumulative variance exceeding the 70% threshold.

| **Principal Component** | **Eigenvalue** | **Explained variance (%)** | **Cumulative variance (%)** |
| --- | --- | --- | --- |
| PC1 | 2.920 | 48.34 | 48.34 |
| PC2 | 1.647 | 27.26 | 75.60 |

**Supplementary Table S2.** Factor loadings of the six PCA-input variables on the two retained principal components. PC1 represents global neuromuscular performance; PC2 represents the mechanical strategy continuum (elastic vs. concentric).

| **Variable** | **PC1 loading** | **PC2 loading** | **PC1 interpretation** | **PC2 interpretation** |
| --- | --- | --- | --- | --- |
| Body mass | -0.330 | 0.167 | Low-moderate (negative) | Low (positive) |
| CMJ jump height | 0.470 | -0.327 | Moderate (positive) | Low-moderate (negative) |
| CMJ modified RSI | 0.445 | -0.130 | Moderate (positive) | Low (negative) |
| DJ inverse contact time | 0.344 | 0.628 | Low-moderate (positive) | High (positive) |
| DJ RSI-ratio | 0.420 | 0.534 | Moderate (positive) | Moderate (positive) |
| SJ jump height | 0.421 | -0.411 | Moderate (positive) | Moderate (negative) |

**Supplementary Table S3.** Pairs of predictor and PCA-input variables with high Pearson correlations (|r| ≥ 0.70). These correlations reflect inherent biomechanical dependencies among variables derived from the same motor task and were used to define the reduced-predictor subset for the sensitivity analysis.

| **Predictor variable** | **PCA-input variable** | **Pearson r** |
| --- | --- | --- |
| DJ peak power | DJ RSI-ratio | 0.943 |
| DJ peak power | DJ inverse contact time | 0.894 |
| SJ peak power | SJ jump height | 0.853 |
| CMJ peak power | CMJ jump height | 0.835 |
| DJ eccentric mean force | DJ inverse contact time | 0.783 |
| CMJ contraction time | CMJ modified RSI | -0.780 |
| SJ concentric mean force | Body mass | 0.762 |
| DJ eccentric mean force | DJ RSI-ratio | 0.731 |
| CMJ peak power | SJ jump height | 0.719 |
| DJ concentric mean force | DJ inverse contact time | 0.717 |

**Supplementary Table S4.** Agreement between the median-based labeling strategy and three alternative unsupervised clustering approaches, quantified using the Adjusted Rand Index (ARI). Values indicate moderate-to-substantial agreement, supporting the robustness of the median-based categorization.

| **Alternative labeling strategy** | **Adjusted Rand Index (ARI)** | **Agreement level** |
| --- | --- | --- |
| K-Means | 0.602 | Substantial |
| Gaussian Mixture Model | 0.404 | Moderate |
| Ward hierarchical clustering | 0.550 | Substantial |

**Supplementary Table S5.** Nested cross-validation results (5-outer × 3-inner StratifiedGroupKFold). Hyperparameter tuning was performed exclusively in the inner loop. The consistency of these estimates with grouped cross-validation confirms that default hyperparameters did not inflate performance.

| **Algorithm** | **F1-Score (mean ± SD)** | **AUC-ROC (mean ± SD)** |
| --- | --- | --- |
| SVM RBF | 0.752 ± 0.070 | 0.930 ± 0.037 |
| Logistic Regression | 0.748 ± 0.090 | 0.932 ± 0.043 |
| Linear SVM | 0.718 ± 0.104 | 0.920 ± 0.041 |
| Random Forest | 0.703 ± 0.055 | 0.907 ± 0.039 |

**Supplementary Table S6.** Reduced-predictor sensitivity analysis. Models were re-trained after excluding the 7 predictors most strongly correlated with PCA-input variables (|r| ≥ 0.70). Logistic Regression retained moderate discriminative capacity, confirming that classification did not depend exclusively on highly correlated variables.

| **Predictor set** | **Algorithm** | **F1-Score** | **AUC-ROC** |
| --- | --- | --- | --- |
| Full (16 predictors) | Logistic Regression | 0.830 | 0.977 |
| Full (16 predictors) | Linear SVM | 0.830 | 0.957 |
| Full (16 predictors) | SVM RBF | 0.810 | 0.958 |
| Full (16 predictors) | Random Forest | 0.615 | 0.930 |
| Reduced (9 predictors) | Logistic Regression | 0.720 | 0.910 |
| Reduced (9 predictors) | Linear SVM | 0.753 | 0.915 |
| Reduced (9 predictors) | SVM RBF | 0.481 | 0.879 |
| Reduced (9 predictors) | Random Forest | 0.421 | 0.822 |

**Supplementary Table S7.** Variable subset ablation by jump test using Logistic Regression. Each row represents a model trained on a specific subset of jump-derived predictors. Removing all DJ-derived variables produced the largest performance drop, confirming the central discriminative role of the drop jump.

| **Predictor subset** | **N predictors** | **F1-Score** | **AUC-ROC** |
| --- | --- | --- | --- |
| All predictors | 16 | 0.830 | 0.977 |
| DJ + SJ | 9 | 0.809 | 0.959 |
| CMJ + DJ | 13 | 0.796 | 0.968 |
| DJ only | 6 | 0.669 | 0.921 |
| CMJ only | 7 | 0.643 | 0.894 |
| CMJ + SJ (Without DJ) | 10 | 0.604 | 0.916 |
| SJ only | 3 | 0.553 | 0.878 |
